# Predicting patient-reported outcome of activities of daily living in stroke rehabilitation: a machine learning study

**DOI:** 10.1186/s12984-023-01151-6

**Published:** 2023-02-23

**Authors:** Yu-Wen Chen, Keh-chung Lin, Yi-chun Li, Chia-Jung Lin

**Affiliations:** 1 School of Occupational Therapy, College of Medicine, National Taiwan University, 17, F4, Xuzhou Rd., Taipei, Taiwan; 2 Division of Occupational Therapy, Department of Physical Medicine and Rehabilitation, National Taiwan University Hospital, 7 Chung-Shan S. Rd., Taipei, Taiwan; 3 Department of Occupational Therapy, I-Shou University College of Medicine, Kaohsiung, Taiwan; 4 Department of Speech Language Pathology and Audiology, National Taipei University of Nursing and Health Sciences, Taipei, Taiwan

**Keywords:** Stroke rehabilitation, Prognosis, Prediction, Patient-reported outcome measures, Activities of daily living, Machine learning

## Abstract

**Background:**

Machine Learning is increasingly used to predict rehabilitation outcomes in stroke in the context of precision rehabilitation and patient-centered care. However, predictors for patient-centered outcome measures for activities and participation in stroke rehabilitation requires further investigation.

**Methods:**

This study retrospectively analyzed data collected for our previous studies from 124 participants. Machine Learning models were built to predict postintervention improvement of patient-reported outcome measures of daily activities (i.e, the Motor Activity Log and the Nottingham Extended Activities of Daily Living) and participation (i.e, the Activities of Daily Living domain of the Stroke Impact Scale). Three groups of 18 potential predictors were included: patient demographics, stroke characteristics, and baseline assessment scores that encompass all three domains under the framework of International Classification of Functioning, Disability and Health. For each target variable, classification models were built with four algorithms, logistic regression, k-nearest neighbors, support vector machine, and random forest, and with all 18 potential predictors and the most important predictors identified by feature selection.

**Results:**

Predictors for the four target variables partially overlapped. For all target variables, their own baseline scores were among the most important predictors. Upper-limb motor function and selected demographic and stroke characteristics were also among the important predictors across the target variables. For the four target variables, prediction accuracies of the best-performing models with 18 features ranged between 0.72 and 0.96. Those of the best-performing models with fewer features ranged between 0.72 and 0.84.

**Conclusions:**

Our findings support the feasibility of using Machine Learning for the prediction of stroke rehabilitation outcomes. The study was the first to use Machine Learning to identify important predictors for postintervention improvement on four patient-reported outcome measures of activities and participation in chronic stroke. The study contributes to precision rehabilitation and patient-centered care, and the findings may provide insights into the identification of patients that are likely to benefit from stroke rehabilitation.

## Background

Stroke is a leading cause of disability that requires long-term post-stroke care and rehabilitation [1]. Along the course, patients and family and the care team are required to make multiple clinical decisions. Clinical decision making in rehabilitation benefits from accurate predictions of prognosis, which prompts research that investigates predictors for stroke-rehabilitation outcomes.

Two recent trends in rehabilitation are precision rehabilitation and patient-centered care. Clinical decision making in the context of precision rehabilitation involves identifying the characteristics of patients who would likely benefit from rehabilitation programs. Machine learning (ML) is increasingly used for the task of understanding predictors for rehabilitation outcomes by the construction of models that can predict outcomes when given new data. ML is a branch of artificial intelligence that uses algorithms to find patterns in the input data and generate models to predict target variables. Through pattern-finding, the models identify the most important “features,” or potential predictors, for the “target,” or the predicted variable. The advantages of ML include its ability to take a large amount of features at once, to conduct multidimensional data analyses, and to learn from the data without substantial a priori knowledge about the features [2].

In stroke rehabilitation, studies have investigated the feasibility of ML models for the prediction of postintervention outcomes. Most studies focused on patients in the subacute stage. The predicted outcome measures in these studies represent the three domains of the World Health Organization’s International Classification of Functioning, Disability and Health (ICF) [3], and range from measures of motor function, including the Ten-Meter Walk Test, Six-Minute Walk Test, and Berg Balance Scale [4], to measures of activities and participation, including the Barthel Index [5, 6], the modified Rankin Scale [7–10], the Functional Independence Measure (FIM) [4], and patients’ discharge placement [11, 12]. However, few ML predictive studies on chronic stroke investigated the postintervention outcomes [13–16]. To our knowledge, two studies investigated postintervention improvements in upper-limb (UL) motor function measured by the Fugl-Meyer Assessment Upper Extremity subscale (FMA-UE) [13, 14] or lower-limb motor function measured by step threshold [16]. One study used the Stroke Impact Scale (SIS), a measure in the ICF domain of activities and participation. Studies using ML remains scarce on the prediction of postintervention improvements, especially in measures of the ICF domains of activities and participation, for chronic stroke.

The other recent trend in medicine and rehabilitation, patient-centered care, aims at engaging the patients, family, and caregivers in the clinical decision-making process. To achieve this goal, patient-reported outcome measures (PROMs) for activities and participation should be incorporated in the assessment in addition to therapist-rated and impairment-level measures. However, most of the existing ML predictive studies on stroke rehabilitation outcomes investigated therapist-rated outcome measures such as the Barthel Index [5, 6] and the FIM [4] for the acute and subacute stages. In the chronic stage, earlier reports studied the FMA-UE [13, 14], and one recent study investigated SIS [15] as the concept of PROMs emerges. There is still a need to expand our knowledge of the relevance of ML predictive models to include more commonly used PROMs of activities and participation.

Another common practice found in the literature has been the inclusion of only one predicted outcome measure. However, given the heterogeneous nature of the stroke population, including multiple predicted outcome measures in research studies was recommended [4]. In fact, most therapists use multiple assessment tools to quantify related but distinct aspects of body functions, activities, and participation in clinical practice. For example, the Motor Activity Log (MAL) [17] and the Nottingham Extended Activities of Daily Living (NEADL) [18, 19] are commonly used patient-reported assessment tools of activities, and the SIS [20] has been widely used to measure function of participation.

The MAL was designed to measure the use of the affected upper-limb in basic activities of daily living (ADL). Patients are asked to rate how much (amount of use; MAL-AOU) and how well (quality of movement; MAL-QOM) they use the affected arm for a number of given ADL. The NEADL measures instrumental ADL and assesses functional independence in community living. The SIS measures patients’ health-related quality of life and includes items for participation; one of its domains is Activities of Daily Living (SIS-ADL). Assessing multiple outcome measures to provide multifaceted clinical information about potential prognosis could empower the patients and their families to make appropriate decisions that are most relevant and meaningful to the patient. However, most predictive studies only reported one outcome measure. There is a need to expand the repertoire of outcome measures in research studies to meet clinical applications.

This study used ML to build predictive models to predict postintervention outcomes and identify the most important predictors for these outcome measures in stroke rehabilitation. We have expanded on previous findings to use multiple PROMs for activities and participation in consideration of clinical applications and recent trends in stroke rehabilitation.

## Methods

### Study design and participants

This study is a retrospective analysis of data collected for previous studies conducted by our research team; available results have been published elsewhere [21, 22]. The inclusion criteria of the original studies were (1) at least 3 months after the onset of a first-ever unilateral cerebral stroke; (2) a baseline FMA-UE between 16 and 56; (3) ability to follow instructions, with one study including only participants without Wernicke’s aphasia; (4) a spasticity score of ≤ 3 on the Modified Ashworth Scale; and (5) no other neurologic or orthopedic disorders. The exclusion criteria of the original studies were (1) serious vision disorders in one study [22] and (2) psychiatric and balance problems in the other study [21].

### Intervention and assessment

The participants received one of the following therapy programs: InMotion robotic-assisted therapy, Bi-Manu-Track robotic therapy [21], robotic-priming mirror therapy, robotic-priming bilateral upper limb training [22], or conventional occupational therapy. Dosages were similar across the therapy programs; participants received 3 weeks of therapy, 3 to 4 days a week, and 60 min a day. Assessments were completed before and after the therapies, and for most participants, at a 3-month follow-up.

### Outcome measures and potential predictors

Participants’ level of ADL was measured by three assessment tools with four PROMs: the MAL-AOU and MAL-QOM, NEADL, and SIS-ADL. For each measure, participants who achieved the minimal clinically important difference (MCID) from pretest to posttest were labeled as responders, and those who did not were labeled as non-responders. For MAL, we adopted an MCID of 0.5 of average change, corresponding to 10% of the rating scale [23–25]. The MCIDs for NEADL total changes and SIS-ADL total changes were 6.1 [18] and 5.9 [26], respectively. For an ML model, the status of response to therapy (i.e., responders versus non-responders) on a given PROM served as the predicted variable, called the “target” in ML terminology.

We included 18 potential predictors, called “features” in ML terminology, in the ML models. The potential predictors can be grouped into three categories: (1) participant demographics: age, sex, and years of education; (2) stroke characteristics: time since stroke, the National Institute of Health Stroke Scale (NIHSS) score, side of hemiplegia, and diagnosis (hemorrhagic or ischemic); and (3) baseline assessment scores: FMA-UE, Box and Block Test (BBT), Wolf Motor Function Test-Time (WMFT-Time), Wolf Motor Function Test-Quality (WMFT-Quality), Chedoke Arm and Hand Activity Inventory (CAHAI), MAL-AOU, MAL-QOM, NEADL, FIM, SIS-Total, and SIS-ADL. The baseline assessment scores were selected to encompass all three domains under the ICF framework: body function, activities, and participation.

### Data analysis

The potential predictors and the target variables were used to build ML models. The objective of the ML programs was to find patterns to classify the samples into responders and non-responders. For each PROM, four ML algorithms were used to find the patterns: logistic regression (LR), k-nearest neighbors (KNN), support vector machine (SVM), and random forest (RF). KNN and SVM were selected because they were frequently reported to yield high performance in existing predictive studies of stroke rehabilitation outcomes [5, 8, 9, 11, 13, 27]. LR was selected as a baseline model to test the predicting capability of a simpler algorithm for our data set. RF was selected to test whether its higher model complexity would benefit the predictions. Using multiple algorithms to construct models and compare performances is also common. One previous study specifically recommended the use of multiple algorithms [13].

In addition to models with all 18 features, in consideration of clinical parsimony, we also built predictive models with the four, five, and six most important features, which we identified by feature selection procedure (see details in the next paragraph). Therefore, for each target variable, 16 models were built (four algorithms x four numbers of features).

**Fig. 1:**
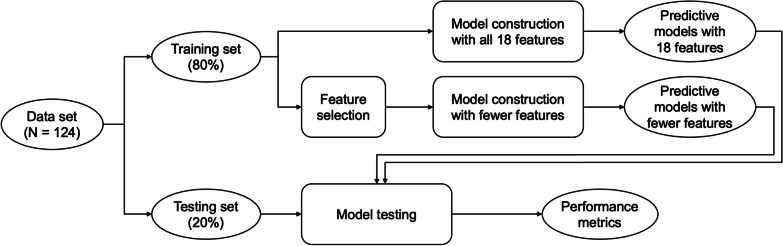
Flowchart for the machine-learning data analysis. *SMOTE* synthetic minority oversampling technique, *KNN* k-nearest neighbors, *SVM* support vector machine

Figure 1 visualizes steps for the data analysis using ML. For each target variable, the data set was first randomized and split into a training set and a testing set, with the training set containing 80% of the samples. The training set was used to build models, and the testing set was used to test the performance of the models. To select the most important features to use in the parsimonious models, feature selection was performed using the standardized training set by calculating mutual information gain (MI; also known as information gain). The testing data set was never used for model construction or feature selection. This ensured that the data used to test model performance did not influence any decisions about the models and was truly unseen until performance testing.

During model construction, the Synthetic Minority Over-sampling Technique (SMOTE) [28] was used to minimize the effect of class imbalance, where models may favor the majority class, creating biases and potential false optimistic classification accuracy. Except for models built with RF, the data were also standardized to avoid dominating effects of features on scales of larger numbers [29]. For model tuning, grid search was used to identify values for hyperparameters that obtained the highest classification accuracy with stratified tenfold cross validation. For LR, the search procedure identified the optimal c value and maximum iterations. For KNN, the search procedure identified the optimal number of neighbors and the distance weight. For SVM, the search procedure identified the optimal kernel and c value, which specifies the size of the hyperplane margin and therefore regularizes the model. For RF, the search procedure identified the optimal number of estimators and maximum depth. All other hyperparameters were set as the default.

After the models were constructed, model performance was tested using the testing set. Model performance was primarily assessed by classification accuracy and the area under the receiver operating characteristic curve (AUC). We also calculated specificity, sensitivity, negative predictive value (NPV), and positive predictive value (PPV).

Descriptive statistics and normality checks were performed with R 4.0.3 software [30]. The construction and validation of the ML models and the corresponding data preprocessing were conducted using Python 3.8.2 software [31], with the packages sklearn 1.0 [32] and imblearn 0.8 [33].

## Results

### Participant characteristics

A total of 128 participants were located in our data base; four participants dropped out before the postintervention assessment, resulting in missing data, and were excluded from the study. The study included 124 participants. Table 1 summarizes the demographics, stroke characteristics, and baseline assessment scores of the participants. Of the 124 participants, 79 achieved MCID for MAL-AOU, 79 for MAL-QOM, 43 for NEADL, and 36 for SIS-ADL.

**Table 1:** Participant Characteristics

	Mean ± SD/ Median (IQR)/ Participants, no. (%)
Demographics	
Age (years)	55.75 ± 11.22
Male sex	84 (68%)
Years of education	12.00 (6.00)
Stroke characteristics	
Right-sided hemiplegia	70 (57%)
Time since stroke (months)	14.00 (22.00)
Hemorrhagic stroke diagnosis	55 (44%)
NIHSS score	4.00 (3.00)
Baseline Assessment Scores	
FMA-UE	30.00 (14.00)
BBT	1.00 (13.25)
CAHAI	31.50 (20.25)
WMFT-Time	12.11 (8.14)
WMFT-Quality	2.41 ± 0.54
FIM	110.00 (12.25)
MAL-AOU	0.91 (1.01)
MAL-QOM	0.59 (0.99)
NEADL	28.00 (25.25)
SIS-Total	63.13 ± 12.04
SIS-ADL	37.50 (10.25)

*SD* standard deviation, *IQR* interquartile range, *NIHSS* National Institutes of Health Stroke Scale, *FMA-UE* Upper Extremity subscale of the Fugl-Meyer Assessment, *BBT* Box and Block Test, *CAHAI* Chedoke Arm and Hand Activity Inventory, *WMFT* Wolf Motor Function Test, *FIM* Functional Independence Measure, *MAL* Motor Activity Log, *AOU* Amount of Use, *QOM* Quality of Movement, *NEADL* Nottingham Extended Activities of Daily Living, *SIS* Stroke Impact Scale, *ADL* Activities of Daily Living

### Most important predictors

Table 2 presents the MI gains for the predictors with gains higher than zero. Notably, across all target variables, their corresponding baseline scores had non-zero MI gains for the achievement of MCID. Further, baseline UL motor function (FMA-UE and BBT) and baseline SIS-Total scores were important for all target variables. MAL scores were also at the top five important predictors for all target variables.

**Table 2:** Mutual information gains for the predictors sorted in descending order for each target variable

MAL-AOU		MAL-QOM		NEADL		SIS-ADL	
Predictor	Gain	Predictor	Gain	Predictor	Gain	Predictor	Gain
Time since stroke	0.14	SIS-Total	0.12	SIS-Total	0.07	FMA-UE	0.10
WMFT-Quality	0.13	WMFT-Quality	0.06	SIS-ADL	0.06	SIS-ADL	0.09
SIS-Total	0.10	MAL-QOM	0.06	NEADL	0.05	MAL-AOU	0.05
FMA-UE	0.06	FMA-UE	0.03	MAL-AOU	0.03	CAHAI	0.05
MAL-QOM	0.06	MAL-AOU	0.03	BBT	0.02	Diagnosis	0.06
CAHAI	0.04	FIM	0.03	NIHSS	0.02	SIS-Total	0.02
FIM	0.03	Side of hemiplegia	0.02	FIM	0.01	Time since stroke	0.01
WMFT-Time	0.03	NEADL	0.02	Other predictors	0	NIHSS	0.01
MAL-AOU	0.01	Sex	0.01			Other predictors	0
Other predictors	0	Years of education	0.01				
		Other predictors	0				

*ADL* Activities of Daily Living, *AOU* Amount of Use, *BBT* Box and Block Test, *CAHAI* Chedoke Arm and Hand Activity Inventory, *FMA-UE* Upper Extremity subscale of the Fugl-Meyer Assessment, *MAL* Motor Activity Log, *NEADL* Nottingham Extended Activities of Daily Living, *NIHSS* National Institutes of Health Stroke Scale, *QOM* Quality of Movement, *SIS* Stroke Impact Scale, *WMFT* Wolf Motor Function Test

### Model performance

**Fig. 2:**
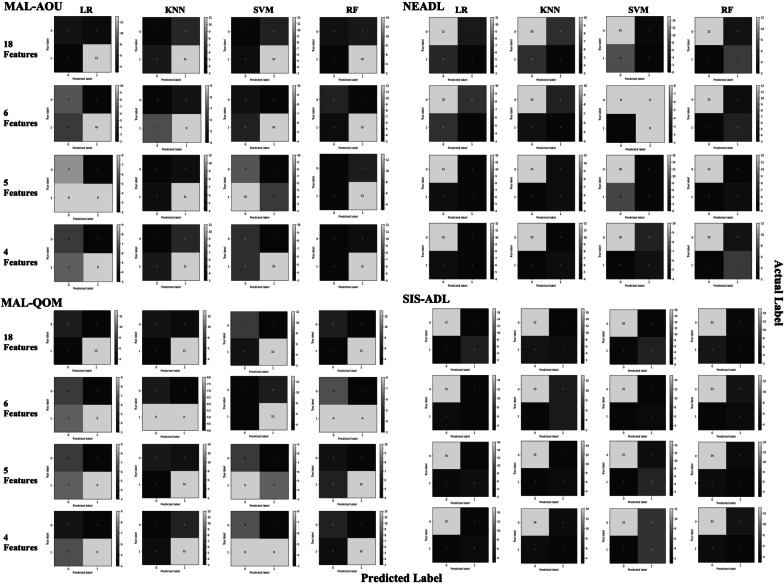
Confusion matrices for the predictive models. *MAL* Motor Activity Log, *AOU* Amount of Use, *QOM* Quality of Movement, *NEADL* Nottingham Extended Activities of Daily Living, *SIS-ADL* Stroke Impact Scale Activities of Daily Living domain, *LR* logistic regression, *KNN* k-nearest neighbors, *SVM* support vector machine, *RF* random forest

Figure 2 visualizes the confusion matrices for the models. Table 3 summaries the performance metrics as well as training scores and medians and interquartile ranges of the validation scores. Good model performance was achieved across the outcome measures. For all outcome measures, similar or slightly decreased prediction accuracies could be achieved with a reduced number of features. Among the MAL-AOU models with 18 features, LR yielded the best performance (accuracy = 0.72, AUC = 0.74). For MAL-AOU models with fewer features, RF with 6 features performed the best (accuracy = 0.72, AUC = 0.80). For MAL-QOM models with 18 features, SVM and RF yielded the best performance (accuracy = 0.76, AUC = 0.83), and LR achieved similar performance (accuracy = 0.76, AUC = 0.81). Among the MAL-QOM models with fewer features, KNN with 5 features performed the best (accuracy = 0.76, AUC = 0.75). For NEADL models with 18 features, RF yielded the best performance (accuracy = 0.76, AUC = 0.81). For NEADL models with fewer features, the best performance occurred with RF fitted with 4 features (accuracy = 0.76, AUC = 0.87). For SIS-ADL predicted with 18 features, SVM yielded the best performance (accuracy = 0.96, AUC = 0.96). For SIS-ADL models fitted with fewer features, SVM with 5 features yielded the best performance (accuracy = 0.84, AUC = 0.92).

**Table 3:** Model performance metrics and training and validation scores for the predictive models

Model	Accuracy	AUC	Specificity	Sensitivity	NPV	PPV	Train score	Validation, median (IQR)
MAL-AOU								
18 features								
LR	0.72	0.74	0.56	0.81	0.63	0.76	0.66	0.60 (0.19)
KNN	0.56	0.48	0.33	0.69	0.38	0.65	0.75	0.60 (0.17)
SVM	0.60	0.56	0.33	0.75	0.43	0.67	0.86	0.63 (0.10)
RF	0.68	0.76	0.44	0.88	0.67	0.74	1.00	0.65 (0.18)
6 features								
LR	0.68	0.74	0.78	0.63	0.54	0.83	0.59	0.47 (0.28)
KNN	0.52	0.66	0.44	0.56	0.36	0.64	1.00	0.65 (0.18)
SVM	0.60	0.69	0.56	0.63	0.45	0.71	0.89	0.65 (0.25)
RF	0.72	0.80	0.67	0.75	0.60	0.80	1.00	0.70 (0.20)
5 features								
LR	0.60	0.69	0.78	0.50	0.47	0.80	0.57	0.58 (0.25)
KNN	0.60	0.56	0.44	0.69	0.44	0.69	1.00	0.60 (0.16)
SVM	0.52	0.77	0.78	0.38	0.41	0.75	0.57	0.50 (0.18)
RF	0.64	0.69	0.33	0.81	0.50	0.68	1.00	0.70 (0.09)
4 features								
LR	0.60	0.70	0.67	0.56	0.46	0.75	0.58	0.53 (0.28)
KNN	0.56	0.60	0.33	0.69	0.38	0.65	1.00	0.65 (0.19)
SVM	0.64	0.74	0.67	0.63	0.50	0.77	0.61	0.60 (0.15)
RF	0.64	0.64	0.44	0.75	0.50	0.71	1.00	0.74 (0.18)
MAL-QOM								
18 features								
LR	0.76	0.81	0.67	0.81	0.67	0.81	0.75	0.68 (0.10)
KNN	0.72	0.78	0.56	0.81	0.63	0.76	0.78	0.50 (0.19)
SVM	0.76	0.83	0.78	0.75	0.64	0.86	0.77	0.68 (0.10)
RF	0.76	0.83	0.67	0.81	0.67	0.81	1.00	0.50 (0.19)
6 features								
LR	0.60	0.71	0.67	0.56	0.46	0.75	0.63	0.60 (0.26)
KNN	0.52	0.57	0.56	0.50	0.38	0.67	0.73	0.65 (0.10)
SVM	0.64	0.49	0.33	0.81	0.50	0.68	0.97	0.60 (0.10)
RF	0.52	0.67	0.67	0.50	0.43	0.73	0.81	0.60 (0.19)
5 features								
LR	0.60	0.72	0.67	0.56	0.46	0.75	0.61	0.60 (0.15)
KNN	0.76	0.75	0.56	0.88	0.71	0.78	1.00	0.60 (0.26)
SVM	0.52	0.62	0.67	0.44	0.40	0.70	0.81	0.60 (0.26)
RF	0.60	0.71	0.56	0.63	0.45	0.71	0.85	0.70 (0.16)
4 features								
LR	0.56	0.72	0.56	0.56	0.42	0.69	0.67	0.60 (0.19)
KNN	0.60	0.62	0.33	0.75	0.43	0.67	1.00	0.70 (0.23)
SVM	0.60	0.71	0.67	0.50	0.43	0.73	0.77	0.70 (0.10)
RF	0.72	0.75	0.67	0.75	0.60	0.80	0.99	0.70 (0.21)
NEADL								
18 features								
LR	0.56	0.57	0.69	0.33	0.65	0.38	0.62	0.50 (0.26)
KNN	0.52	0.41	0.63	0.33	0.63	0.33	0.97	0.60 (0.25)
SVM	0.60	0.65	0.94	0.00	0.63	0.00	0.67	0.65 (0.10)
RF	0.76	0.81	0.75	0.78	0.86	0.64	0.81	0.70 (0.16)
6 features								
LR	0.52	0.57	0.63	0.33	0.63	0.33	0.60	0.65 (0.20)
KNN	0.56	0.48	0.63	0.44	0.67	0.40	0.95	0.60 (0.20)
SVM	0.64	0.62	0.50	0.89	0.89	0.50	0.55	0.70 (0.18)
RF	0.72	0.85	0.75	0.67	0.80	0.60	0.80	0.70 (0.16)
5 features								
LR	0.64	0.72	0.75	0.44	0.71	0.50	0.62	0.65 (0.20)
KNN	0.64	0.63	0.69	0.56	0.73	0.50	0.94	0.60 (0.10)
SVM	0.64	0.76	1.00	0.00	0.64	0.00	0.70	0.60 (0.09)
RF	0.68	0.82	0.75	0.56	0.75	0.56	0.86	0.68 (0.18)
4 features								
LR	0.64	0.72	0.75	0.44	0.71	0.50	0.65	0.60 (0.20)
KNN	0.68	0.71	0.75	0.56	0.75	0.56	0.93	0.68 (0.20)
SVM	0.60	0.70	0.63	0.56	0.71	0.45	0.62	0.60 (0.28)
RF	0.76	0.87	0.75	0.78	0.86	0.64	0.80	0.70 (0.18)
SIS-ADL								
18 features								
LR	0.92	0.98	0.94	0.86	0.94	0.86	0.98	0.90 (0.08)
KNN	0.80	0.75	0.94	0.43	0.81	0.75	0.96	0.68 (0.10)
SVM	0.96	0.96	1.00	0.86	0.95	1.00	0.95	0.90 (0.15)
RF	0.68	0.76	0.83	0.29	0.75	0.40	1.00	0.70 (0.09)
6 features								
LR	0.72	0.80	0.83	0.43	0.79	0.50	0.77	0.75 (0.27)
KNN	0.72	0.77	0.72	0.71	0.87	0.50	0.78	0.70 (0.06)
SVM	0.76	0.82	0.83	0.57	0.83	0.57	0.77	0.70 (0.18)
RF	0.68	0.72	0.72	0.57	0.81	0.44	0.87	0.65 (0.19)
5 features								
LR	0.80	0.81	0.89	0.57	0.84	0.67	0.75	0.65 (0.19)
KNN	0.76	0.76	0.83	0.57	0.83	0.57	0.73	0.65 (0.10)
SVM	0.84	0.92	0.83	0.86	0.94	0.67	0.72	0.70 (0.13)
RF	0.68	0.74	0.78	0.43	0.78	0.43	0.80	0.65 (0.16)
4 features								
LR	0.76	0.87	0.83	0.57	0.83	0.57	0.75	0.70 (0.09)
KNN	0.68	0.69	0.78	0.43	0.78	0.43	0.82	0.70 (0.08)
SVM	0.72	0.88	0.67	0.86	0.92	0.50	0.72	0.74 (0.20)
RF	0.64	0.72	0.72	0.43	0.76	0.38	0.90	0.70 (0.19)

*IQR* interquartile range, *MAL* Motor Activity Log, *AOU* Amount of Use, *QOM* Quality of Movement, *NEADL* Nottingham Extended Activities of Daily Living, *SIS-ADL* Stroke Impact Scale Activities of Daily Living domain, *LR* logistic regression, *KNN* k-nearest neighbors, *SVM* support vector machine, *RF* random forest, *AUC* area under the receiver operating characteristic curve, *NPV* negative predictive value, *PPV* positive predictive value

## Discussion

ML is increasingly used in the prediction of postintervention prognosis in stroke. Previous studies have investigated prognostic predictors as well as the performance of predictive models. However, most studies were on acute to subacute stroke, and few studies exist on chronic stroke. Further, studies on postintervention improvements in subacute stroke included measures of motor function and measures of activities and participation, whereas few studies on chronic stroke investigated activities and participation. In addition, most studies have included only one predicted outcome measure and focused on therapist-rated measures. The use of PROMs is attracting more attention in recent years as health care shifts toward patient-centered care, but few studies have investigated postintervention improvements measured by PROMs in chronic stroke.

This current study extended from the existing literature by investigating the most important predictors for MCID achievements on multiple PROMs for activities and participation in chronic stroke using ML. We identified different sets of the most important predictors for the target variables, reflecting the distinct, albeit related, aspects of ADL assessed in the four PROMs. We also obtained good model performances for the target variables, demonstrating the feasibility of ML for predicting postintervention improvement on PROMs of activities and participation in chronic stroke. In addition, we were able to build parsimonious models with smaller sets of predictors that performed similar or just slightly worse than the full models, which could benefit clinical practice in the selection of prioritized assessments.

### ML for predicting postintervention outcomes in stroke

Emerging research has reported the feasibility of ML for the prediction of postintervention outcome in stroke. However, in the field of health care research, achieving the sample size of big data analysis is often difficult. This is because of a variety of limitations, such as patient privacy policies, the heterogeneity of disease manifestation, the variability in care plans, and cost and time for intervention and data collection, to name just a few. Findings of this current study, however, indicates the feasibility of using ML for the prediction of postintervention outcome with a limited sample size. Despite the relatively smaller sample size, we were able to obtain high classification accuracies and acceptable to outstanding [34] AUCs using techniques to lower the effects of dimensionality and class imbalance.

The practice of feature selection contributed to clinical parsimony. Clinically, it is more efficient if accurate prediction of prognosis can be obtained by assessment results from fewer tools. In our results, at least one of the models with fewer features for each target variable was able to achieve similar performance compared with 18 features. The results provided support for the clinical application of ML by finding that highly accurate predictions of postintervention outcomes in stroke can be achieved with only a few clinical assessments and patient information.

Another issue working with our data set was class imbalance, where one of the classes (responders versus non-responders) outnumbered the other. In a data set with imbalanced classes, the learning machine may focus on finding patterns in the majority class when striving to increase classification accuracy. This usually results in a bias toward the majority class [29]. Consider an extreme example, where there are 90 cases in the positive class and 10 in the negative class, the classifier could conveniently classify all cases as positive and obtain a high training accuracy of 0.90. However, the specificity and NPV would be zero. Among the techniques to work with class imbalance, we chose to use SMOTE because of our relatively smaller sample size (N = 124). Tozlu et al. [14] also used SMOTE to deal with class imbalance; of their 102 participants, 43 achieved MCID on FMA-UE and 59 did not.

### The most important predictors

Our results showed that the baseline scores of a given PROM were among the important features for classifying responders versus non-responders on that measure. This was similar to findings of predictive studies of UL motor function in chronic stroke using ML [13, 14] and traditional statistical methods [35]. In studies for the acute and subacute stages, similar findings have been reported with measures for motor function and ADL.

Iwamoto et al. [36] found that FMA-UE scores at the initiation of inpatient rehabilitation were the most important predictor for identifying participants that would achieve an MCID on the FMA-UE 30 days after treatment. Harari et al. [4] built ML models to predict discharge scores of FIM, Ten-Meter Walk Test, Six-Minute Walk Test, and the Berg Balance Scale after inpatient rehabilitation stay; they found that the most important predictors for these scores were their own scores at admission. In two other studies with patients admitted to inpatient rehabilitation facilities, the discharge Barthel Index scores and improvements were both predicted by the admission Barthel Index scores [5, 6].

Lin et al. [8] analyzed data from a nation-wide disease registry and built predictive models for 90-day post-stroke scores on the modified Rankin Scale. They found that the 30-day modified Rankin Scale scores was the most important predictor for both ischemic and hemorrhagic stroke. Our findings and previous findings together suggest that it is important to include the baseline score of an assessment as a potential predictor in future studies on postintervention outcome prediction.

Baseline UL motor function, namely, the BBT and the FMA-UE, were found to be important predictors for achieving the MCID on all target variables. The finding was consistent with existing literature. In chronic stroke, baseline FMA-UE was found to predict postintervention UL motor function in two studies using ML [13, 14]. For studies using traditional statistical analysis, baseline BBT was found to predict postintervention outcomes of activities and participation [37, 38], and FMA-UE was found to predict both UL motor function and activities and participation [25, 36, 39]. Our findings further supported the predictive value of UL motor function for postintervention achievement of MCID in the PROMs of activities and participation in chronic stroke. Similar findings were also reported for studies using acute and subacute parameters to predict discharge assessment scores or long-term outcomes [5, 6, 40–43]. The similar findings across disease stages suggested that preintervention UL motor function is an important predictor for postintervention outcomes for all stages in stroke and should be included as a potential predictor if available in future predictive studies.

Demographic and stroke characteristics were frequently included as potential predictors in rehabilitation outcome prediction. For example, in chronic stroke, age was previously reported as a predictor for postintervention UL motor function [14] and UL activity [39]. In acute to subacute stroke, age was found to predict the possibility of home discharge after rehabilitation stay [11] and functional outcomes at discharge [6, 44, 45], at 3 months post-stroke [9], and at 6 months post-stroke [10]. Sex has also been previously reported as an important predictor for long-term post-stroke functional outcome [10] and postintervention UL activity [37]. Our results identified only sex and years of education in the lists of predictors with non-zero gains for MAL-QOM. Although the gains were negligibly small at 0.01, indicating their minimal relationship with postintervention achievement of MCID in MAL-QOM, the findings were partially in line with previous studies.

Stroke characteristics, i.e., time since stroke, side of hemiplegia, NIHSS scores, and diagnosis (i.e., hemorrhagic or ischemic) were identified as important predictors for one or two target variables. Previous studies also reported that time since stroke predicted functional outcomes in the subacute stage [4, 44] and postintervention UL motor function for the chronic stage [13, 14]. Stroke severity was previously reported to predict long-term post-stroke functional outcomes in acute and subacute stroke [46–48]; our results showed that stroke severity, as measured by NIHSS, can also predict postintervention improvements in NEADL in chronic stroke. The finding should be cautiously interpreted, however, because there may be an underrepresentation of severe cases in our study. Our participants had NIHSS scores ranging from 0 to 13, which correspond to no stroke symptoms, minor stroke, and moderate stroke. Therefore, this finding should not be generalized to patients with severe stroke in the chronic stage. In summary, our results that demographic and stroke characteristics were among the most important predictors were largely consistent with previous findings, and we recommend future studies include these characteristics in the potential predictors when performing feature selection.

Note that, methodologically, feature selection could be conducted before model construction from the cohort or after model construction for specific models. This study identified the most important predictors a priori from the cohort, instead of post hoc from specific models. This decision took in considerations of the steps adopted by previous studies in stroke rehabilitation [10, 13, 14], clinical applications to identify a set of assessments to prioritize regardless of chosen algorithms, and the reduction of overall complexity of this study.

### Predictive models and predictors across the four PROMs

Despite some overlapping predictors for the four target variables, the four sets of predictors were different. We chose these assessments because they include items for different aspects of activities and participation. There is also a hierarchy among these assessments. The MAL assesses the more basic ADL. The NEADL considers mobility and community living activities. The SIS-ADL considers the daily activities of higher complexities, and some of the activities may require the collaboration of other body parts and/or use of instruments. The important predictors for each target variable likely reflected what each particular assessment tool captures. The findings highlight the importance of using different sets of predictors for these ADL assessments and support the use of feature selection to screen for the most relevant and meaningful predictors in future studies.

Among the four PROMs, postintervention achievement of MCID in SIS-ADL appeared to be predicted well across algorithms and numbers of features used. On the contrary, MCID achievement in NEADL required the more complex method, RF, to achieve good prediction performance. The NEADL concerns mobility and community living activities in an extended context, and may involve aspects not as well captured by the predicting variables we used. Regardless, good prediction performance was achievable with the combination of a more complex prediction method and predictors that cover a wider range of aspects, such as general stroke severity (NIHSS) and overall impact of the stroke (SIS). These measures include items for cognition, mobility, and emotion, among others, that may contribute to the extended aspects of activities and participation.

### Study limitations

The major limitation of this study is the limited sample size; however, we have made an effort to minimize model bias and variance that could result from it by using SMOTE, reducing dimensionality through feature selection, and ensuring that the data used to test model performances did not affect model construction. Through these efforts, we were able to construct at least one model with acceptable to excellent metrics for each target variable. In fact, low specificity and/or sensitivity are commonly seen in the literature using ML to predict stroke rehabilitation outcomes with relatively small sample sizes.

Although we would recommend future studies use larger sample sizes, achieving the size of big data in health care is often difficult and/or costly. Future studies may use more advanced techniques to minimize the effects of small sample sizes.

Further, the accurate prediction of postintervention ADL outcomes may be more complex and involve predictors that were not included in this study. For example, nutritional status [49], aphasia [50, 51], and cognition [52] were reported to predict ADL outcomes after stroke rehabilitation. This study, as a secondary data analysis, did not collect data on all potential predictors, making it impossible to address these predictors. Future studies may investigate the predictive power of a wider range of predictors when investigating postintervention ADL in the stroke population.

Finally, ML is characterized by its data-driven nature, and therefore the results of this study, as well as many other studies using ML, may not be readily generalized to data from other facilities or other patient characteristics. However, this study and previous studies have repeatedly confirmed the feasibility of ML in predicting postintervention outcomes in the stroke population. Further, some predictors were repeatedly reported and may be important to consider in future studies, such as UL motor function, selected demographic and stroke characteristics, and baseline scores of assessments used to quantify the outcomes. We recommend that health care facilities develop their own models by taking findings of this and previous studies as references.

## Conclusion

In this study, we obtained high accuracies and AUCs using ML to predict postintervention PROMs for activities and participation in chronic stroke, demonstrating the feasibility of ML methods for this research task. We also identified the most important predictors for achieving MCID on these PROMs. Consistent with existing literature, UL motor function, selected demographic and stroke characteristics, and the baseline scores of the PROMs were important predictors across the four PROMs. Individual predictors identified for the PROMs also reflected the characteristics and contexts of the ADL that these assessments capture. The study findings may contribute to precision rehabilitation by providing insights into the identification of patients that are likely to benefit from stroke rehabilitation.

## Data Availability

The data sets used and/or analyzed during the current study are available from the corresponding author on reasonable request.

## Abbreviations

ADL: Activities of daily living
AUC: Area under the receiver operating characteristic curve
BBT: Box and Block Test
CAHAI: Chedoke Arm and Hand Activity Inventory
FIM: Functional Independence Measure
FMA-UE: Upper Extremity subscale of the Fugl-Meyer Assessment
ICF: International Classification of Functioning, Disability and Health
KNN: K-nearest neighbors
LR: Logistic regression
MAL-AOU: Motor Activity Log Amount of Use
MAL-QOM: Motor Activity Log Quality of Movement
MCID: Minimal clinically important difference
MI: Mutual information
ML: Machine learning
NEADL: Nottingham Extended Activities of Daily Living
NIHSS: National Institute of Health Stroke Scale
NPV: Negative predictive value
PPV: Positive predictive value
PROM: Patient-reported outcome measure
RF: Random forest
SIS: Stroke Impact Scale
SIS-ADL: Activities of Daily Living domain of the Stroke Impact Scale
SMOTE: Synthetic Minority Over-sampling Technique
SVM: Support vector machine
UL: Upper limb
WMFT: Wolf Motor Function Test
